# A Case Report of Syphilis That Was Difficult to Distinguish From Penile Carcinoma

**DOI:** 10.7759/cureus.45029

**Published:** 2023-09-11

**Authors:** Masayuki Tomioka, Hiromi Uno, Kensaku Seike, Koji Iinuma, Miyazaki Tatsuhiko

**Affiliations:** 1 Urology, Chuno Kousei Hospital, Seki, JPN; 2 Urology, Gifu University Graduate School of Medicine, Gifu, JPN; 3 Pathology, Gifu University Graduate School of Medicine, Gifu, JPN

**Keywords:** treponema pallidum, lymph node biopsy, massive lymphadenopathy, metastatic penile cancer, syphilis

## Abstract

A 43-year-old man presented with penile induration and lymphadenopathy. Computed tomography revealed multiple enlarged lymph nodes (LNs). Penile cancer was suspected, and a LN biopsy was performed. Histopathological examination revealed inflammation and fibrosis, with no findings indicating malignancy. Serological examination confirmed syphilis and treatment with amoxicillin was initiated. Thereafter, swelling in the LNs improved quickly. Penile cancer is usually suspected in the presence of penile induration. However, syphilis can also present with similar symptoms. To distinguish between syphilis and penile cancer, the patient's history, results of physical examination, and presence of tumor and infectious markers should be considered.

## Introduction

Syphilis is a sexually transmitted infection caused by the bacterium Treponema pallidum. If not treated properly, the disease can progress and occasionally even be fatal [1]. Since its historic low in 2000-2001, the prevalence of syphilis has been increasing annually, especially in 2020-2021, when a 28.6% increase was observed [2]. Moreover, syphilis infection rates continue to rise worldwide, with 176,713 cases reported in 2021 [2]. In Japan, the prevalence of syphilis is expected to increase, and several cases have been difficult to diagnose because of the atypical symptoms in the early stages of infection. Herein, we report a case in which it was difficult to differentiate between penile cancer and syphilis.

## Case presentation

**Figure 1:**
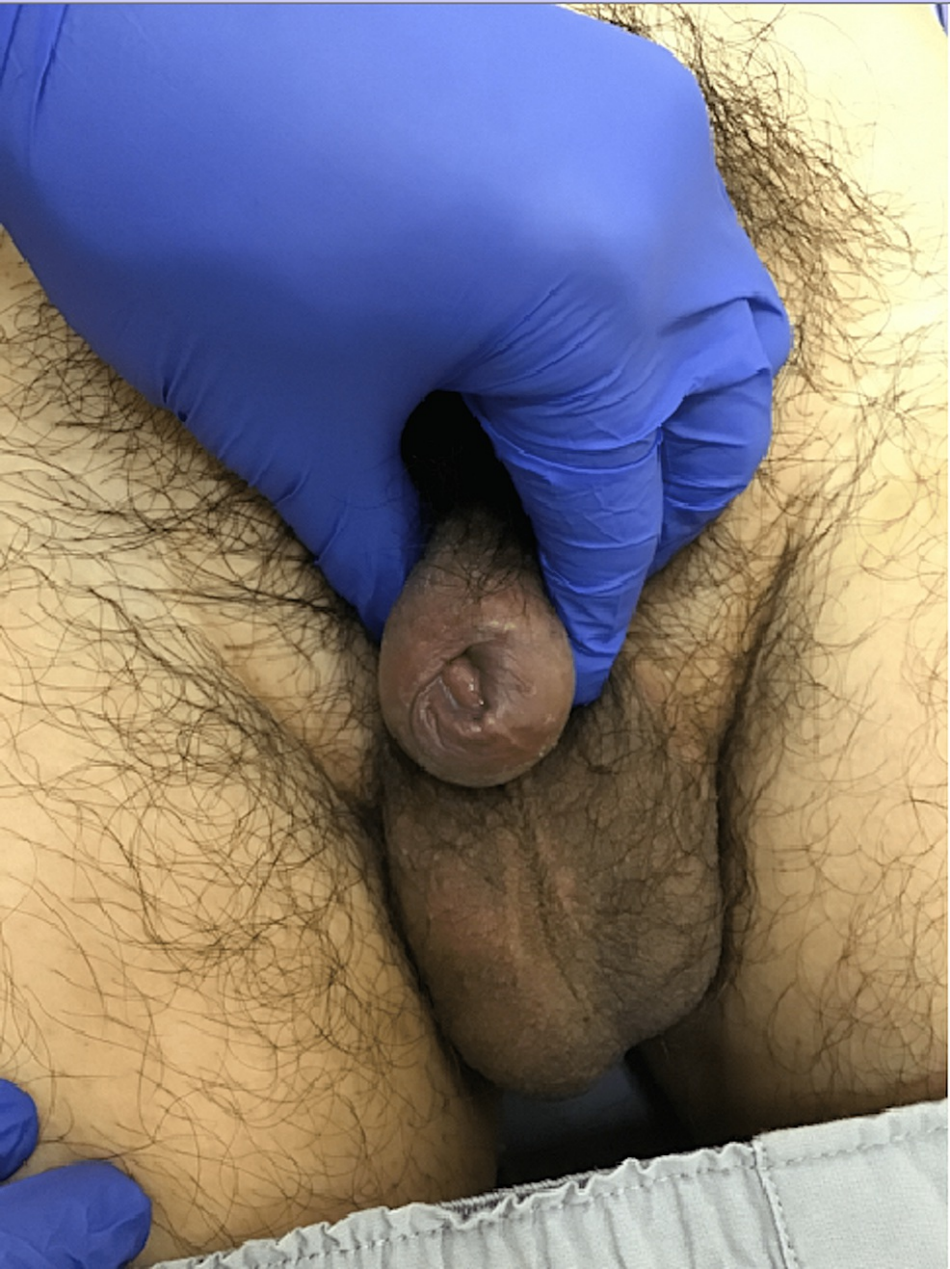
Penile foreskin Painless induration of the penile foreskin and its firm adhesion to the penis are observed.

A 43-year-old man presented to our hospital with a chief complaint of itching of the penile foreskin. On physical examination, a 30 mm painless induration was palpated on the penile foreskin, approximately 5 mm from the external urethral orifice with the presence of phimosis (Figure 1).

**Figure 2:**
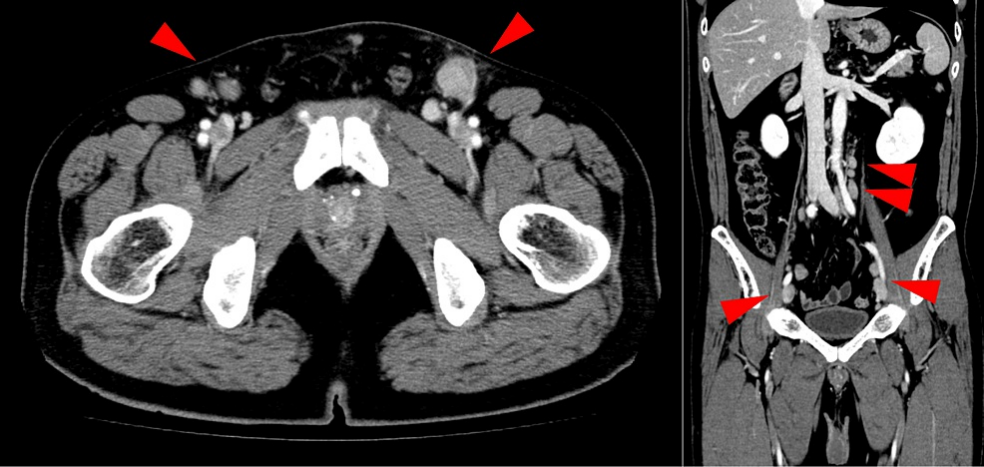
Computed tomography Contrast-enhanced computed tomography showing multiple enlarged lymph nodes in the bilateral inguinal, pelvic, and para-aortic regions taking up contrast (red arrowhead).

Bilateral inguinal lymph nodes were also palpable. However, no ulceration or redness was observed in the pubic region. Additionally, the foreskin was adherent to the penis, even though induration was observed on the penis. Therefore, we could not confirm the skin rash at the initial examination and could not diagnose syphilis. Computed tomography revealed multiple enlarged lymph nodes, including the bilateral inguinal, pelvic, and para-aortic lymph nodes. The left inguinal lymph node was the largest with a diameter of 40 mm (Figure 2).

**Figure 3:**
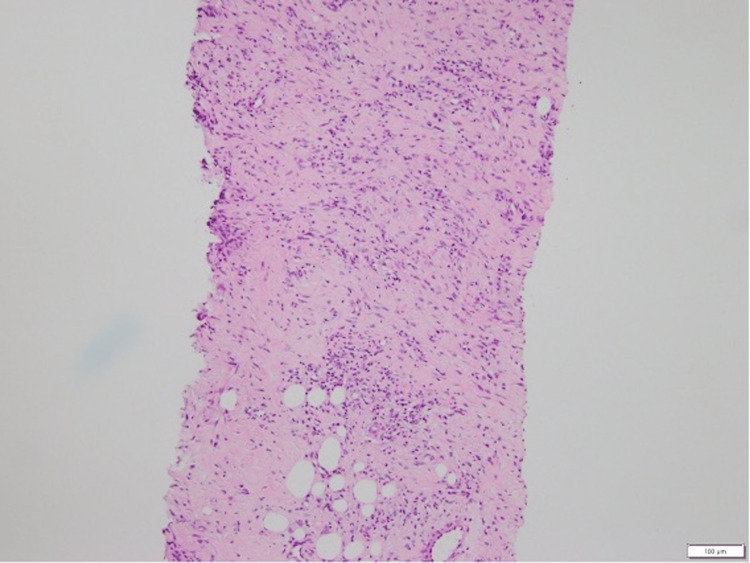
Needle biopsy specimen from the left inguinal lymph node The tissue sample obtained from the left inguinal lymph node through a needle biopsy shows only inflammation and fibrosis on hematoxylin and eosin staining, with no findings of malignancy.

Penile carcinoma was suspected, even though the squamous cell carcinoma antigen level was only 1.3 ng/mL, and a needle biopsy of the left inguinal lymph node was performed. A penile biopsy was attempted; however, it could not be performed due to the difficulty of penile exposure as a result of adhesions between the foreskin and the penis. Histopathological examination of the biopsied specimen revealed only inflammation and fibrosis, with no evidence of malignancy (Figure 3).

Therefore, the patient was interviewed again in detail; this time he disclosed that he had visited a sex establishment three months earlier. The rapid plasma reagin test (RPR), as a non-treponemal test, and the Treponema pallidum hemagglutination assay (TPHA), as a treponemal test, were both positive. Thus, the patient was diagnosed with syphilis, with multiple enlarged lymph nodes secondary to the infection. Tests for sexually transmitted infections, including human immunodeficiency virus, gonorrhea, and chlamydia, were negative. A daily dose of 1.5 g of oral amoxicillin was initiated. After 28 days of initiation of treatment for syphilis, the patient showed a reduction in swelling of multiple lymph nodes and improvement in induration, adhesion, and itching of the penile foreskin. Furthermore, the serum RPR level had reduced from 67.2 U/mL at the time of diagnosis to 3.7 U/mL after treatment with amoxicillin.

## Discussion

In this case, a penile carcinoma was initially suspected because a detailed examination revealed the presence of multiple enlarged lymph nodes, including bilateral inguinal lymph nodes. However, the lymph node biopsy revealed no malignancy. Subsequently, the patient was diagnosed with syphilis based on the positive RPR and TPHA results and treated with oral amoxicillin, which reduced the lymph node swelling. Benzathine penicillin G (2.4 million units intramuscularly), the standard therapy for syphilis, has only recently been approved in Japan. Therefore, this case was treated with oral amoxicillin [3].

Syphilis progresses in stages, with the early stages characterized by the formation of chancres at the site of infection, especially on the genitalia [1]. In the second stage, a rash, fever, and systemic lymphadenopathy develops. Syphilis advances to the third stage after several years, causing damage to the heart, brain, and nervous system. Treatment involves penicillin, which is considered most effective in the early stages of infection [1]. However, if left untreated, syphilis can lead to serious health complications including blindness, paralysis, and death. It is important to remember that it may be challenging to distinguish syphilis from penile carcinoma in cases of initial infection and atypical clinical symptoms, such as those in the present patient. Penile cancer and syphilis both can present with symptoms, such as penile induration and swollen lymph nodes [1] and may lead to delays in appropriate treatment and management. Moreover, gummatous tumors, a sign of tertiary syphilis, have been occasionally misdiagnosed as penile and testicular tumors [4,5]. The rapid improvement in symptoms after amoxicillin administration, as seen in our case, suggests that syphilis is a curable disease and that early intervention with antibiotics may markedly improve patient outcomes. Therefore, syphilis should be considered as a differential diagnosis of malignant penile neoplasms in the presence of penile induration or a history of rash, fever, or sexually transmitted infection.

In differentiating syphilis from penile cancer and determining the appropriate treatment, diagnostic tools such as blood tests and tissue biopsies play an important role. Additionally, in the histopathological examination of the biopsied specimens, immunohistochemical staining using anti-trepenomal antibodies is reportedly useful for the diagnosis of syphilis [6]. Therefore, obtaining as much tissue as possible, including penile and lymph node specimens, and using the aforementioned diagnostic method could help differentiate syphilis from penile cancer. For this purpose, a careful physical examination and elicitation of a more detailed history are important.

## Conclusions

The case report suggests that differentiating between penile cancer and syphilis can be difficult, as patients can present with similar symptoms. Although blood tests and tissue biopsies can assist in making an accurate diagnosis, a detailed history and physical examination is imperative. Syphilis is a disease where early intervention with antibiotics can significantly improve patient outcomes. Therefore, in view of the increasing number of cases, syphilis should be considered as a differential diagnosis for penile cancer, in the presence of penile induration.
